# Physical Activity, Alzheimer Plasma Biomarkers, and Cognition

**DOI:** 10.1001/jamanetworkopen.2025.0096

**Published:** 2025-03-05

**Authors:** Seung Ae Kim, Daeun Shin, Hongki Ham, Yeshin Kim, Yuna Gu, Hee Jin Kim, Duk L. Na, Henrik Zetterberg, Kaj Blennow, Sang Won Seo, Hyemin Jang

**Affiliations:** 1 Department of Neurology, Seoul National University Hospital, Seoul, South Korea; 2 Seoul National University College of Medicine, Seoul, South Korea; 3 Department of Neurology, Samsung Medical Center, Sungkyunkwan University School of Medicine, Gangnam-gu, Seoul, South Korea; 4 Alzheimer’s Disease Convergence Research Center, Samsung Medical Center, Seoul, South Korea; 5 Department of Health Sciences and Technology, SAIHST, Sungkyunkwan University, Seoul, South Korea; 6 Department of Digital Health, SAIHST, Sungkyunkwan University, Seoul, South Korea; 7 Department of Intelligent Precision Healthcare Convergence, Sungkyunkwan University, Suwon, South Korea; 8 Department of Neurology, Kangwon National University College of Medicine, Chuncheon, South Korea; 9 Happymid Clinic, Seoul, South Korea; 10 Department of Psychiatry and Neurochemistry, Institute of Neuroscience and Physiology, The Sahlgrenska Academy at the University of Gothenburg, Gothenburg, Sweden; 11 Clinical Neurochemistry Laboratory, Sahlgrenska University Hospital, Gothenburg, Sweden; 12 Department of Neurodegenerative Disease, University College London Institute of Neurology, Queen Square, London, United Kingdom; 13 UK Dementia Research Institute, University College London, London, United Kingdom; 14 Hong Kong Center for Neurodegenerative Diseases, Hong Kong, China; 15 Wisconsin Alzheimer’s Disease Research Center, University of Wisconsin School of Medicine and Public Health, University of Wisconsin–Madison, Madison; 16 Paris Brain Institute, ICM, Pitié-Salpêtrière Hospital, Sorbonne University, Paris, France; 17 Neurodegenerative Disorder Research Center, Division of Life Sciences and Medicine, and Department of Neurology, Institute on Aging and Brain Disorders, University of Science and Technology of China and First Affiliated Hospital of USTC, Hefei, China

## Abstract

**Importance:**

Physical activity (PA) is a nonpharmacological intervention for dementia prevention. The association between PA and Alzheimer disease (AD) plasma biomarkers remains underexplored.

**Objective:**

To investigate the associations among PA; plasma biomarkers, including β-amyloid 42/40 (Aβ42/40), phosphorylated-tau217 (ptau217), glial fibrillary acidic protein (GFAP), and neurofilament light chain (NfL); and cognition.

**Design, Setting, and Participants:**

This cross-sectional study included participants with and without cognitive impairment recruited from multiple memory clinics in South Korea between May 2019 and May 2022. Data were analyzed from June to December 2024.

**Exposures:**

PA was assessed as metabolic equivalent task minutes per week using the International Physical Activity Questionnaire and categorized into quartiles from the lowest (Q1) to the highest (Q4).

**Main Outcomes and Measures:**

Plasma Aβ42/40, ptau217, GFAP, and NfL were measured. Cognition was assessed using the Mini-Mental State Examination (MMSE) and Clinical Dementia Rating-Sum of Boxes (CDR-SB).

**Results:**

Among 1144 participants (mean [SD] age 70.9 [8.7] years; 744 [65.0%] female), the highest PA quartile showed significantly lower ptau217 (estimate [SE], −0.14 [0.06]; *P* = .01) and NfL (estimate [SE], −0.12 [0.05]; *P* = .01) compared with the lowest quartile. Higher PA quartiles were associated with higher MMSE scores (estimate [SE]: Q2, 0.93 [0.31]; *P* = .003; Q3, 0.82 [0.32]; *P* = .009; Q4, 0.94 [0.32]; *P* = .004) and lower CDR-SB scores (estimate [SE]: Q2, –0.33 [0.16]; *P* = .04; Q3, –0.37 [0.16]; *P* = .02; Q4, –0.55 [0.16]; *P* = .001) after adjusting for age, sex, education years, and β-amyloid uptake. In subgroup analyses according to age and cognitive status, the associations of PA and plasma biomarkers with cognition were more pronounced in the older (age ≥65 years) and cognitively impaired groups compared with the younger and cognitively unimpaired groups.

**Conclusions and Relevance:**

These findings suggest that PA may help delay cognitive decline by modulating neurodegeneration and AD-specific tau pathologies. However, the cross-sectional design limits causal inference, and longitudinal studies are needed to confirm and clarify these associations.

## Key Points

**Question:**

Is physical activity (PA) associated with Alzheimer disease (AD) plasma biomarkers and cognition?

**Findings:**

In this cross-sectional study of 1144 participants, including individuals with and without cognitive impairment, higher levels of PA were significantly associated with lower levels of plasma neurofilament light chain and phosphorylated tau-217 and better cognition. These associations were more pronounced in the cognitively impaired group and the group aged 65 years and older compared with the cognitively unimpaired and younger groups.

**Meaning:**

These findings suggest that PA might be linked to protection against neurodegeneration, AD pathology, and cognitive impairment.

## Teaser

This cross-sectional study examines associations among physical activity, plasma biomarkers, and cognition in a multicenter cohort of patients with and without cognitive impairment.

## Introduction

Alzheimer disease (AD),^1,2^ the most common cause of cognitive impairment, is characterized by β-amyloid (Aβ) plaques and tau neurofibrillary tangles, which lead to neuroinflammation, neurodegeneration, and cognitive decline.^3,4^ Although recent Aβ-targeting therapies offer hope,^5^ the complex nature of AD requires additional pharmacological and nonpharmacological interventions to prevent or delay cognitive impairment.

Among various interventions, physical activity (PA) is widely recommended.^1^ Numerous studies suggest that PA not only slows cognitive decline but also improves specific cognitive functions.^2,6,7,8^ Potential mechanisms underlying the protective associations of PA with cognition include its influence on neuroplasticity, neuroinflammation, and cerebral blood flow.^9,10,11,12,13^ Additionally, several animal studies demonstrated that PA can lower brain Aβ and tau,^2,14^ although these findings were less compelling in human studies.^14,15,16,17^ These inconsistencies highlight the need for larger, more comprehensive studies to better understand the impact of PA on AD biomarkers. Furthermore, although PA could potentially affect various pathophysiological mechanisms, few studies have investigated the entire spectrum of biomarkers in relation to PA. Thus, analyzing the relationships among PA, biomarkers for multiple pathomechanisms, and cognition could provide insights into how PA impacts cognitive health.

Recent advancements in plasma biomarkers have advanced diagnostic accuracy and the ability to estimate prognosis in neurodegenerative diseases.^18,19,20,21,22,23,24,25^ The Aβ42:40 ratio and phosphorylated-tau (p-tau) species are regarded as core biomarkers of AD pathology.^26^ Additionally, glial fibrillary acidic protein (GFAP) and neurofilament light chain (NfL) have been studied as promising plasma biomarkers reflecting neuroinflammation^27,28^ and neuronal injury, respectively.^29,30^ Research has shown that these plasma biomarkers are also associated with cognitive decline.^31,32,33,34^ Thus, plasma biomarkers could be potential therapeutic targets, and identifying modifiable factors, such as PA, that influence these biomarkers is crucial for developing management strategies to mitigate cognitive decline.

Thus, this study aimed to examine the associations of PA and plasma biomarkers with cognition using a large multicenter study conducted within memory clinics. Identifying these associations would provide insights into the pathomechanisms underlying the cognitive benefits of PA.

## Methods

This cross-sectional study was approved by the institutional review board at each participating center and adhered to the ethical guidelines specified in the Declaration of Helsinki. All participants provided written informed consent for the study. The study adheres with the Strengthening the Reporting of Observational Studies in Epidemiology (STROBE) reporting guideline.

### Study Participants

Participants were recruited from a large multicenter study, the Precision Medicine Platform for Mild Cognitive Impairment Based on Multi-omics, Imaging, Evidence-Based R&BD (PREMIER) Consortium,^35^ that involved 25 centers across South Korea. As a nationally funded study, participants (and/or caregivers) who visited memory clinics and met the inclusion criteria were asked to volunteer for the study, without selection bias, to undergo all assessments, including detailed questionnaires, neuropsychological tests, blood sampling, and Aβ PET scans. Exclusion criteria included inadequate visual or auditory acuity for neuropsychological assessments, psychiatric diagnoses, active cancer diagnosis, unstable medical conditions, history of neurosurgery, or subcortical vascular cognitive impairment indicated by severe white matter hyperintensities on magnetic resonance imaging. Further details are in the eMethods in Supplement 1.

**Figure 1.**
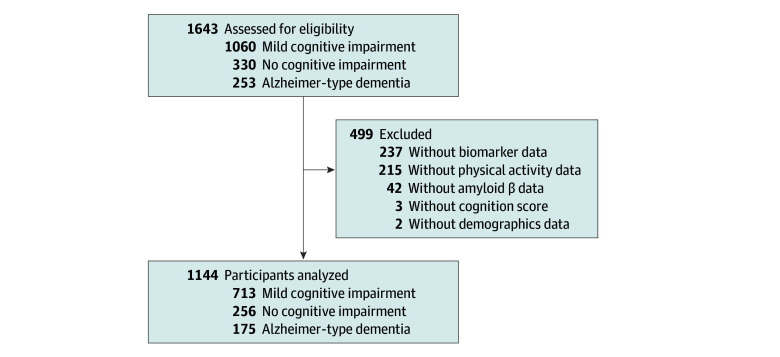
Flowchart of Study Participants

From May 2019 to May 2022, 1643 participants were recruited, including those who were cognitively unimpaired^36^ and cognitively impaired, including amnestic mild cognitive impairment^37,38^ or those with Alzheimer-type dementia.^39^ However, 499 participants (eTable 1 in Supplement 1) were excluded from the study due to incomplete data, resulting in a final sample of 1144 participants (256 participants without cognitive impairment and 888 participants with cognitive impairment) (Figure 1).

### Quantification of Aβ Uptakes

Participants underwent either 18F-florbetaben (FBB) or 18F-flutemetamol (FMM) positron emission tomography (PET) scans, following the manufacturer’s protocols. Specifically, a 20-minute emission PET scan in dynamic mode (consisting of 4 × 5-minute frames) was performed 90 minutes after the administration of a mean dose of 311.5 MBq for FBB or 197.7 MBq for FMM. The PET images were reconstructed in a 128 × 128 × 48 matrix with a voxel size of 2 × 2 × 3.27 mm using the ordered-subset expectation maximization algorithm (FBB and FMM: iteration = 4; subset = 20). The regional direct comparison centiloid method was used to measure Aβ uptake on FBB and FMM PET scans.^40^

### PA Assessment

PA was evaluated using the International Physical Activity Questionnaire.^41^ This tool measured the duration (time spent per day) and frequency (days per week) of different PA types, such as vigorous and moderate activities, walking, and sedentary behavior, over the past 7 days. The collected data were subsequently converted into metabolic equivalent task–minutes per week following the guidelines (eMethods in Supplement 1).^41^ For participants with cognitive impairment who were unable to comprehend or complete the questionnaire independently, PA levels were assessed through caregiver-reported responses.

### Plasma Biomarkers and Cognitive Assessment

Plasma was separated from the blood drawn in a 0.5M EDTA-containing tube and aliquoted into vials of 0.3 mL. Plasma samples were stored at –75 °C until analysis, and frozen plasma samples were transported at –70 °C to the Department of Psychiatry and Neurochemistry, University of Gothenburg, for analysis. Plasma levels of GFAP and NfL were measured using the Neurology 4-Plex E kit (Quanterix; Billerica), while p-tau217 levels were measured using the ALZpath p-tau217 assay kit. Further details on the sample collection and processing are provided in the eMethods in Supplement 1. For cognitive outcomes, Mini-Mental State Examination (MMSE)^42^ and the Clinical Dementia Rating-Sum of Boxes (CDR-SB)^43^ were conducted by trained neuropsychologists.

### Statistical Analysis

Total metabolic equivalent task–minutes per week, a measurement variable of PA, was categorized into quartiles (Qs) due to the skewed data distribution: Q1 (33.0 to <594.0), Q2 (594.0 to <1386.0), Q3 (1386.0 to <2747.3), and Q4 (2747.3 to 15930.0). An analysis of variance was used to compare the clinical characteristics of PA quartile groups. Plasma biomarker values were log2-transformed owing to the nonnormality of the data. Multivariable linear regression was conducted to investigate the associations between PA and plasma biomarkers. We used 3 models to account for potential confounders. Model 1 adjusted for age and sex. Model 2 further adjusted for Aβ uptake (represented by global centiloid), and model 3 additionally adjusted for hypertension, diabetes, body mass index, and estimated glomerular filtration rate,^44,45^ which could potentially affect plasma biomarker levels. Definitions of variables are in the eMethods in Supplement 1. We adjusted for Aβ uptake in the analyses, as Aβ is a well-recognized factor that directly influences plasma biomarker levels and to evaluate the independent association between PA and plasma biomarkers. To investigate the associations between PA and cognition, multivariable linear regression was performed adjusting for age, sex, Aβ uptake, and education years. For cognition, Aβ uptake was similarly adjusted to account for its potential confounding effect and to examine the independent relationship between PA and cognition.

We explored plasma biomarkers as a potential mediator of the association between PA (exposure) and cognition (outcome). Although cross-sectional design limits the interpretation of mediation analyses, we hypothesized that PA may influence plasma biomarkers and subsequently cognition and set the temporal ordering of variables in the mediation analyses. Covariates included age, sex, years of education, and Aβ uptakes. The natural indirect effect (NIE) and natural direct effect (NDE) of PA on cognition were estimated. All statistical analyses were performed using R software version 4.4.1 (R Project for Statistical Computing). Statistical significance was defined as 2-sided *P* < .05. Data were analyzed from June to December 2024.

## Results

### Demographics and Baseline Characteristics

Demographic and clinical characteristics of the PA groups are presented in Table 1. The mean age differed across the groups, with the Q4 group being the youngest (mean [SD]: Q1, 71.8 [9.4] years; Q2, 71.7 [8.2] years; Q3, 71.2 [8.7] years; Q4, 68.9 [8.2]; *P* < .001). The proportion of female participants was lowest in the Q4 group (Q1, 213 participants [74.5%]; Q2, 207 participants [72.4%]; Q3, 177 participants [61.9%]; Q4, 147 participants [51.4%]; *P* < .001). The education level, cognitive status, and Aβ uptake (represented as global regional direct comparison centiloid) did not differ across groups (Table 1).

**Table 1.** Demographic and Clinical Characteristics of Study Participants

Characteristic	Participants, No (%), physical activity quartile (N = 1144)^a^				*P* value
	1	2	3	4	
Age, mean (SD), y	71.8 (9.4)	71.7 (8.2)	71.2 (8.7)	68.9 (8.2)	<.001
Sex					
Male	73 (25.5)	79 (27.6)	109 (38.1)	139 (48.6)	<.001
Female	213 (74.5)	207 (72.4)	177 (61.9)	147 (51.4)	
Education, y	10.3 (4.9)	10.5 (4.5)	11.2 (4.7)	10.9 (4.6)	.06
Diagnosis					
Cognitively unimpaired	50 (17.5)	71 (24.8)	73 (25.5)	62 (21.7)	.14
MCI	181 (63.3)	173 (60.5)	176 (61.5)	183 (64)	
DAT	55 (19.2)	42 (14.7)	37 (12.9)	41 (14.3)	
Vascular risk factors					
Hypertension	143 (50)	144 (50.3)	138 (48.3)	137 (47.9)	.92
Diabetes	72 (25.2)	75 (26.2)	56 (19.6)	219 (76.6)	.26
eGFR, mean (SD)	88.4 (16.1)	88.8 (14.2)	88.2 (15.4)	88.2 (15.1)	.95
BMI, mean (SD)	23.6 (3.4)	23.7 (3.8)	23.8 (3.2)	23.3 (2.5)	.34
Aβ uptake, mean (SD)^b^	50.5 (57.9)	48.1 (54.2)	43.4 (53.9)	40.4 (53.9)	.12

Abbreviations: Aβ, β-amyloid; BMI, body mass index (calculated as weight in kilograms divided by height in meters squared); MCI, mild cognitive impairment; DAT, dementia of the Alzheimer type; eGFR, estimated glomerular filtration rate.

^a^ Quartiles are measured as metabolic equivalent task minutes per week indicating total physical activity levels, calculated from the International Physical Activity Questionnaire, with the first quartile representing the lowest level of physical activity and the fourth, the highest.

^b^ Measured as regional direct conversion centiloid.

### Association of PA With Plasma Biomarkers

Table 2 presents the association between PA and plasma biomarkers. In fully adjusted models, Aβ42:40 ratio was not associated with PA. For ptau217, only the Q4 group demonstrated a significant negative association compared with the Q1 group (estimate [SE], −0.14 [0.06]; *P* = .01). For GFAP, no quartiles showed significant associations. For NfL, the Q3 and Q4 groups showed significant negative associations with NfL compared with Q1 (estimate [SE]: Q3, −0.10 [0.05]; *P* = .047; Q4, −0.12 [0.05]; *P* = .01). The trend analysis indicated significant associations between PA quartiles and both ptau217 and NfL in all models.

**Table 2.** Association Between Physical Activity, Plasma Biomarkers, and Cognition

Biomarker	Physical activity quartile^a^							*P* value for trend
	1	2		3		4		
		Estimate (SE)	*P* value	Estimate (SE)	*P* value	Estimate (SE)	*P* value	
**Aβ42:40 ratio**								
Crude	0 [Reference]	−0.02 (0.03)	.60	0.03 (0.03)	.35	0.04 (0.03)	.20	.09
Model 1^b^	0 [Reference]	−0.02 (0.03)	.62	0.04 (0.03)	.28	0.05 (0.04)	.20	.09
Model 2^c^	0 [Reference]	−0.02 (0.03)	.44	0.02 (0.03)	.59	0.02 (0.03)	.50	.30
Model 3^d^	0 [Reference]	−0.02 (0.03)	.47	0.02 (0.03)	.52	0.03 (0.03)	.44	.26
**Ptau217**								
Crude	0 [Reference]	−0.10 (0.10)	.30	−0.20 (0.10)	.04	−0.34 (0.10)	<.001	<.001
Model 1^b^	0 [Reference]	−0.10 (0.10)	.28	−0.19 (0.10)	.045	−0.27 (0.10)	.005	.003
Model 2^c^	0 [Reference]	−0.06 (0.06)	.27	−0.08 (0.06)	.16	−0.14 (0.06)	.01	.02
Model 3^d^	0 [Reference]	−0.05 (0.05)	.35	−0.05 (0.05)	.31	−0.14 (0.06)	.01	.01
**GFAP**								
Crude	0 [Reference]	−0.01 (0.07)	.95	−0.04 (0.07)	.59	−0.28 (0.07)	<.001	<.001
Model 1^b^	0 [Reference]	0.001 (0.06)	.99	0.005 (0.06)	.93	−0.15 (0.06)	.02	.03
Model 2^c^	0 [Reference]	0.02 (0.05)	.73	0.05 (0.05)	.30	−0.09 (0.05)	.09	.17
Model 3^d^	0 [Reference]	0.03 (0.05)	.57	0.07 (0.05)	.18	−0.10 (0.05)	.06	.13
**NfL**								
Crude	0 [Reference]	−0.09 (0.06)	.14	−0.16 (0.06)	.009	−0.26 (0.06)	<.001	<.001
Model 1^b^	0 [Reference]	−0.09 (0.05)	.08	−0.15 (0.05)	.006	−0.15 (0.06)	.01	.003
Model 2^c^	0 [Reference]	−0.09 (0.05)	.10	−0.13 (0.05)	.01	−0.13 (0.05)	.01	.009
Model 3^d^	0 [Reference]	−0.07 (0.05)	.14	−0.10 (0.05)	.047	−0.12 (0.05)	.01	.01
**MMSE**								
Crude	0 [Reference]	1.06 (0.37)	.004	1.33 (0.37)	<.001	1.43 (0.37)	<.001	<.001
Adjusted^e^	0 [Reference]	0.93 (0.31)	.003	0.82 (0.32)	.009	0.94 (0.32)	.004	.007
**CDR-SB**								
Crude	0 [Reference]	−0.37 (0.18)	.04	−0.47 (0.18)	.008	−0.59 (0.18)	.001	<.001
Adjusted^e^	0 [Reference]	−0.33 (0.16)	.04	−0.37 (0.16)	.02	−0.55 (0.16)	.001	.001

Abbreviations: Aβ, β-amyloid; CDR-SB, clinical dementia rating-sum of boxes; GFAP, glial fibrillary acidic protein; MMSE, Mini-Mental State Examination; NfL, neurofilament light chain; ptau217, phosphorylated tau 217.

^a^ Quartiles are measured as metabolic equivalent task minutes per week indicating total physical activity levels, calculated from the International Physical Activity Questionnaire, with the first quartile representing the lowest level of physical activity and the fourth, the highest.

^b^ Adjusted for age and sex.

^c^ Further adjusted for Aβ uptakes.

^d^ Further adjusted for hypertension, diabetes, estimated glomerular filtration rate, and body mass index.

^e^ Adjusted for age, sex, education years, and Aβ uptakes.

### Association of PA With Cognition

Compared with the Q1 group, all quartiles demonstrated higher MMSE (estimate [SE]: Q2, 0.93 [0.31]; *P* = .003; Q3, 0.82 [0.32]; *P* = .009); Q4, 0.94 [0.32]; *P* = .004) and lower CDR-SB (estimate [SE]: Q2, –0.33 [0.16]; *P* = .04; Q3, –0.37 [0.16]; *P* = .02; Q4, −0.55 [0.16]; *P* = .001) after adjusting for covariates. The trend was statistically significant, indicating that higher levels of PA were associated with better MMSE (*P* for trend = .007) and lower CDR-SB *(P* for trend = .001) (Table 2).

### Subgroup Analyses According to Age and Cognitive Status

We further analyzed the data according to age and cognitive status (Table 3). Age was categorized into 876 participants aged 65 years or older and 268 participants younger than 65 years. Demographic comparisons between age groups are shown in eTable 2 in Supplement 1. In the older age group, significant associations were observed between PA and plasma biomarkers, including ptau217, GFAP, and NfL. In contrast, no significant associations between PA and plasma biomarkers were observed in the younger age group. The association between PA and cognition remained significant in the older age group, as all higher quartiles (Q2, Q3, and Q4) demonstrated significantly better MMSE and CDR-SB scores than the reference group (Q1). In contrast, these associations were less pronounced in the younger age group. Higher PA quartiles (Q2, Q3, and Q4) did not exhibit statistically significant associations with MMSE scores compared with Q1, although Q2 and Q3 were associated with lower CDR-SB scores.

**Table 3.** Subgroup Analyses According to Cognitive Status and Age

Subgroup	Physical activity quartile^a^							*P* value for trend
	1	2		3		4		
		Estimate (SE)	*P* value	Estimate (SE)	*P* value	Estimate (SE)	*P* value	
**Age <65 y**								
Aβ42:40 ratio^b^	0 [Reference]	−0.07 (0.06)	.25	0.04 (0.06)	.51	0.03 (0.06)	.59	.26
GFAP^b^	0 [Reference]	0.13 (0.11)	.25	0.08 (0.11)	.48	−0.03 (0.10)	.78	.55
Ptau217^b^	0 [Reference]	0.03 (0.11)	.79	−0.11 (0.11)	.33	−0.06 (0.10)	.55	.36
NfL^b^	0 [Reference]	−0.03 (0.10)	.80	−0.15 (0.10)	.14	−0.08 (0.10)	.42	.31
MMSE^c^	0 [Reference]	0.97 (0.81)	.23	1.16 (0.82)	.16	0.65 (0.82)	.43	.42
CDR-SB^c^	0 [Reference]	−1.23 (0.47)	.01	−1.09 (0.48)	.02	−0.86 (0.48)	.07	.12
**Age ≥65 y**								
Aβ42:40 ratio^b^	0 [Reference]	−0.01 (0.04)	.77	0.02 (0.04)	.65	0.02 (0.04)	.64	.51
GFAP^b^	0 [Reference]	0.004 (0.06)	.95	0.07 (0.06)	.21	−0.12 (0.06)	.045	.18
Ptau217^b^	0 [Reference]	−0.06 (0.06)	.31	−0.03 (0.06)	.59	−0.16 (0.06)	.01	.03
NfL^b^	0 [Reference]	−0.07 (0.05)	.18	−0.08 (0.06)	.17	−0.13 (0.06)	.02	.03
MMSE^c^	0 [Reference]	1.02 (0.37)	.006	1.12 (0.37)	.003	1.16 (0.38)	.002	.003
CDR-SB^c^	0 [Reference]	−0.31 (0.18)	.09	−0.50 (0.18)	.006	−0.58 (0.18)	.002	.001
**Cognitively unimpaired**								
Aβ42:40 ratio^b^	0 [Reference]	−0.01 (0.06)	.84	0.08 (0.06)	.20	0.03 (0.07)	.62	.32
GFAP^b^	0 [Reference]	0.02 (0.10)	.85	0.11 (0.10)	.26	−0.03 (0.10)	.75	.99
Ptau217^b^	0 [Reference]	−0.13 (0.10)	.17	−0.10 (0.09)	.30	−0.20 (0.10)	.04	.08
NfL^b^	0 [Reference]	−0.04 (0.09)	.69	−0.14 (0.09)	.10	−0.05 (0.09)	.61	.38
MMSE^c^	0 [Reference]	0.11 (0.30)	.71	0.12 (0.31)	.70	0.01 (0.31)	.97	.96
CDR-SB^c^	0 [Reference]	0.02 (0.09)	.83	−0.20 (0.09)	.02	−0.10 (0.09)	.29	.08
**Cognitively impaired**								
Aβ42:40 ratio^b^	0 [Reference]	−0.02 (0.04)	.50	0.001 (0.04)	.99	0.03 (0.04)	.48	.40
GFAP^b^	0 [Reference]	0.03 (0.06)	.55	0.07 (0.06)	.26	−0.12 (0.06)	.045	.10
Ptau217^b^	0 [Reference]	−0.03 (0.06)	.67	−0.03 (0.06)	.61	−0.14 (0.06)	.04	.045
NfL^b^	0 [Reference]	−0.09 (0.06)	.11	−0.07 (0.06)	.25	−0.15 (0.06)	.009	.02
MMSE^c^	0 [Reference]	0.59 (0.40)	.14	0.70 (0.40)	.08	1.06 (0.41)	.01	.01
CDR-SB^c^	0 [Reference]	−0.29 (0.21)	.17	−0.40 (0.22)	.07	−0.74 (0.22)	<.001	<.001

Abbreviations: Aβ, β-amyloid; CDR-SB, clinical dementia rating-sum of boxes; GFAP, glial fibrillary acidic protein; MMSE, Mini-Mental State Examination; NfL, neurofilament light chain; ptau217, phosphorylated tau 217.

^a^ Quartiles are measured as metabolic equivalent task minutes per week indicating total physical activity levels, calculated from the International Physical Activity Questionnaire, with the first quartile representing the lowest level of physical activity and the fourth, the highest.

^b^ For biomarkers (Aβ42/40, GFAP, Ptau217, NfL), analyses were adjusted for age, sex, Aβ uptakes, hypertension, diabetes, estimated glomerular filtration rate, and body mass index.

^c^ For cognition scores (MMSE and CDR-SB), analyses were adjusted for age, sex, and education years.

In the cognitively impaired group (888 participants), PA was significantly associated with lower levels of plasma biomarkers (ptau217, GFAP, and NfL) and better cognition (MMSE and CDR-SB). In contrast, these associations were not observed in the cognitively unimpaired group (256 participants), for whom only Q4 showed lower plasma ptau217 levels and Q3 showed better CDR-SB scores compared with Q1. Further results from subgroup analyses, adjusted for various covariates, are presented in eTables 3 through 6 in Supplement 1.

### Associations Among PA, Plasma Biomarkers, and Cognition

Based on the hypothesis that PA influences plasma biomarkers and cognition, we set the PA quartiles (Q4 vs Q1) as an exposure variable. Since PA was associated with only ptau217 and NfL and marginally with GFAP, mediation analyses were conducted for these 3 biomarkers. We confirmed that plasma biomarkers were also associated with MMSE and CDR-SB scores before mediation analyses (eTable 7 in Supplement 1).

**Figure 2.**
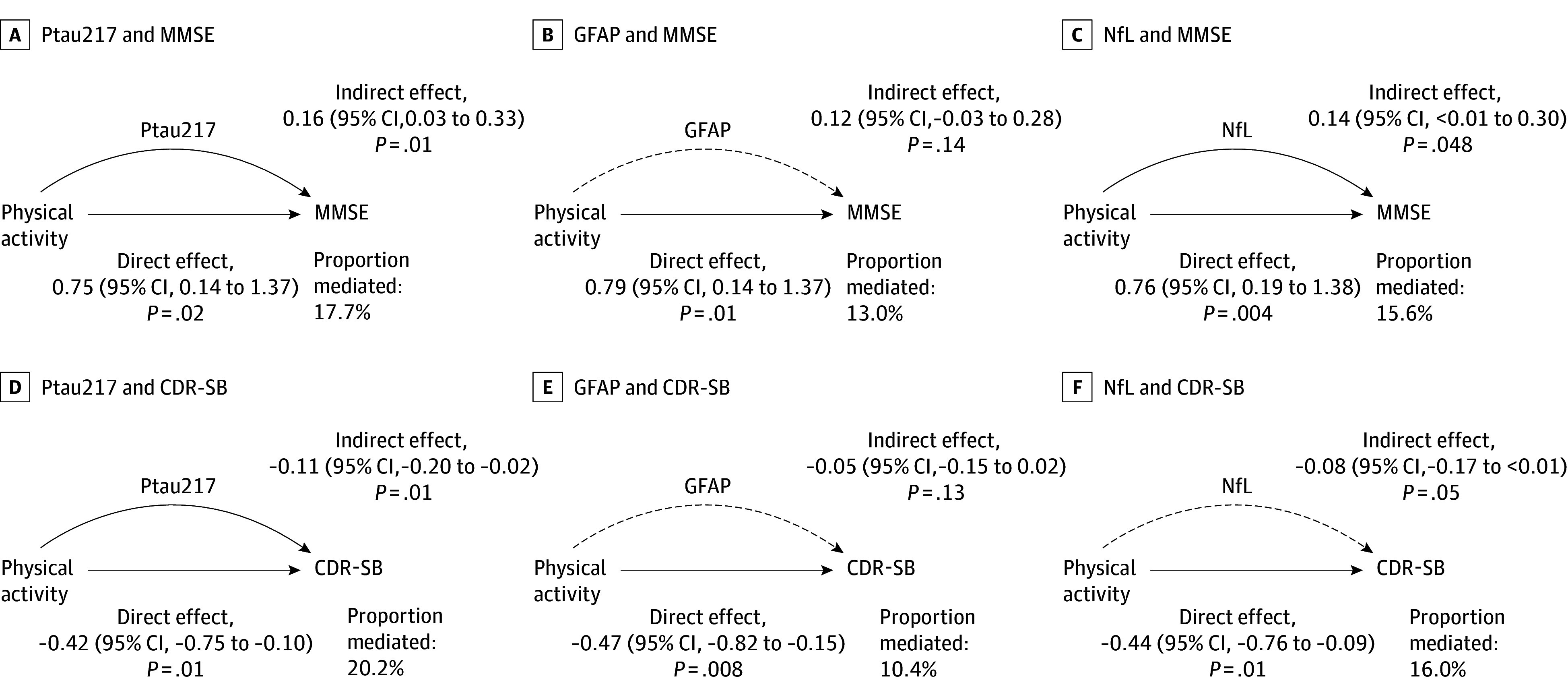
Mediation Analysis of Physical Activity and Cognitive Outcomes Mediation analysis illustrating the association of the highest quartile (vs the lowest quartile) of physical activity with plasma biomarkers and cognitive outcomes as measured by Mini-Mental State Examination (MMSE) and Clinical Dementia Rating-Sum of Boxes (CDR-SB) scores. Solid lines indicate significant associations (*P* < .05); dashed lines, no significant association (*P* ≥ .05). Direct and indirect effects are presented as coefficients with 95% CIs with *P *values. GFAP indicates glial fibrillary acidic protein; NfL, neurofilament light chain; and ptau, phosphorylated tau.

Ptau217 partially mediated the association between PA and cognition. Ptau217 mediated 17.7% of the association of PA with MMSE (NIE, 0.16 [95% CI, 0.03 to 0.33]; *P* = .01) and 20.2% for the association of PA with CDR-SB (NIE, –0.11 [95% CI, –0.20 to –0.02]; *P* = .01). Significant direct associations of PA with MMSE (NDE, 0.75 [95% CI, 0.14 to 1.37]; *P* = .02) and CDR-SB (NDE, –0.42 [95% CI, –0.75 to –0.10]; *P* = .01) were also observed. NfL mediated 15.6% of the association of PA with MMSE (NIE, 0.14 [95% CI, <0.01 to 0.30]; *P* = .048) and but did not significantly mediate the association of PA with CDR-SB (NIE, –0.08 [95% CI, –0.17 to <0.01]; *P* = .05). We observed significant direct associations of PA with MMSE (NDE, 0.76 [95% Cl, 0.19 to 1.38]; *P* = .004) and CDR-SB (NDE, –0.44 [95% CI, –0.76 to –0.09]; *P* = .01). Mediation findings for GFAP were not significant (Figure 2).

## Discussion

This cross-sectional study investigated the associations among PA, plasma biomarkers, and cognition. We had 3 major findings. First, higher levels of PA were associated with lower ptau217 and NfL and better cognition. Second, these associations were more prominent in the older and cognitively impaired groups. Third, the mediation analysis, based on the hypothesis that PA may influence plasma biomarkers and cognition, suggested that PA may impact cognition directly and indirectly through AD pathophysiology. These findings could be used to support the significance of PA in cognition, although future longitudinal studies are needed to disentangle the complex causal relationship.

Our first major finding indicated that the higher level of PA was associated with lower plasma NfL and ptau217 and better cognition. NfL was the biomarker most consistently associated with PA. Although a causal relationship cannot be argued, this finding suggests a possibility of the protective role of PA against neurodegeneration.^46,47^ In fact, there is evidence that higher PA levels are associated with reduced cerebral volume loss,^48^ and individuals who engage more frequently in moderate to vigorous exercise have larger brain volumes, suggesting that PA plays a vital role against neurodegeneration.^12,13^ In terms of the association between PA and NfL, insufficient PA has been reported to be associated with exacerbated age-related degeneration of brain and spinal motor neurons, increasing NfL production.^49,50,51^ However, other intervention studies have reported no significant associations of PA with these biomarkers.^52,53,54^ This might be due to insufficient intervention duration, intensity, or timing of blood sample collection. Therefore, further prospective studies are necessary to establish a causal relationship between PA and NfL. Our study found no significant association between PA and Aβ42:40 ratio or GFAP. It is generally accepted that PA could attenuate neuroinflammatory processes by reducing both systemic and central nervous system inflammation.^55^ Thus, we expected that GFAP, a marker of astrocytic activation, would be correlated with PA.^56,57^ However, previous evidence suggested that GFAP levels increase in response to Aβ aggregation rather than nonspecific neuroinflammation.^58,59^ Therefore, our findings might indicate that PA is not associated with Aβ accumulation. Notably, plasma ptau217 was significantly associated with PA. This finding has important clinical implications, as ptau217 is an AD-specific biomarker independently linked to both Aβ and tau pathologies.^1,60,61^ In our study, a significant association between PA and ptau217 even after adjusting for Aβ uptake on PET suggests that the pathophysiology of AD, particularly tau accumulation independent of Aβ, might be linked to PA. We found that higher PA was associated with better cognition, consistent with previous studies supporting a positive impact of PA on cognition.^2,6,7,8,62,63,64,65^ However, these findings should be interpreted with caution, as participants with worse cognition may tend to be less physically active.

Second, the associations of PA with plasma biomarkers and cognition were more pronounced in the older and cognitively impaired groups compared with the younger and cognitively unimpaired groups. In older adults, PA may directly influence AD pathophysiology and cognition, while its impact may be limited in younger individuals. This suggests that PA’s effects on AD-related processes may require longer durations to manifest, which could explain the lack of significant findings in younger participants. Alternatively, observed associations may reflect greater biological vulnerability in older individuals, such as a weakened blood-brain barrier, making plasma biomarkers more responsive to PA. Given the cross-sectional design, reverse causation is also possible, with only older individuals showing reduced PA due to underlying AD or cognitive impairment. Alternatively, the lack of significant findings in younger participants may also be due to the smaller sample size, limiting statistical power.

In the cognitively impaired group, PA was significantly associated with ptau217, GFAP, and NfL, indicating its association with more advanced AD processes, including tauopathy, glial activation, and neuronal injury. However, determining causality remains challenging in the cognitively impaired group, as individuals with advanced disease burden might be physically inactive. Meanwhile, in the CU group, PA was negatively associated with only ptau217, suggesting a potential protective role in early AD pathologies. Given that the cognitively unimpaired group did not exhibit overt cognitive impairment, this result supports the interpretation that PA may play a protective role in early AD pathologies rather than reflecting reverse causation.

Finally, the mediation analyses based on the hypothetical ordering of variables found that plasma NfL and ptau217 partially mediated the associations of PA with cognition, as represented by MMSE scores. Thus, PA may improve cognition by delaying neurodegeneration and tau pathophysiology, independent of Aβ burden.^46,47^ In addition, PA may directly enhance cognitive and functional performance through mechanisms beyond those captured by plasma biomarkers. These mechanisms could include improved cardiovascular health, increased cerebral blood flow, enhanced neuroplasticity, and better management of mood or physical frailty.^9,10,11,12,13^ This underscores the multifaceted impact of PA on cognitive and functional health, emphasizing its role as a crucial factor in managing cognitive decline and supporting daily life activities.

This study has several strengths. The inclusion of a large, multicenter cohort, comprising 1144 participants, enhances the generalizability of the study and minimizes the risk of biases arising from single-center studies. Additionally, our study used plasma biomarkers as outcome measures, which are anticipated to be integrated into clinical practice as therapeutic targets for future PA interventions.

### Limitations

This study had some limitations that should be acknowledged. First, the cross-sectional nature represents associations rather than causal relationships. Particularly, we used mediation analyses based on a hypothesis; however, this approach has notable limitations in a cross-sectional design and requires confirmation through longitudinal studies. Second, we relied on self-reported questionnaires, which are prone to recall bias and measurement errors. For individuals with cognitive impairment, caregiver-reported data were obtained based on clinical judgments regarding the participant’s comprehension, which may vary among clinicians. Additionally, variations in caregiver familiarity with the participant’s daily activities and their ability to accurately recall PA levels may introduce bias or errors. Third, while we adjusted for potential confounders, unmeasured confounders may still influence the observed associations. Furthermore, our study was conducted only in South Korea, so our findings may not be generalizable to populations with different ethnic or cultural backgrounds. Therefore, further research in diverse populations is required to evaluate the broader applicability of our results.

## Conclusions

In this cross-sectional study, PA was associated with lower plasma ptau217 and NfL levels and better cognition, suggesting a potential role in delaying cognitive decline by modulating neurodegeneration and AD pathophysiology. However, the cross-sectional design limits causal inference, and longitudinal studies are required to confirm these findings and clarify the direction of these associations.

## Notes

Supplement 1.
eMethods.
**eTable 1.** Demographic Comparison Between Included and Excluded Participants **eTable 2.** Comparison Between Younger and Older Age Groups **eTable 3.** Association Between Physical Activity and Plasma Biomarkers in the Old Age (≥65) Group **eTable 4.** Association Between Physical Activity and Biomarkers in the Younger Age (<65) Group **eTable 5.** Association Between Physical Activity and Plasma Biomarkers in the Cognitively Unimpaired Group **eTable 6.** Association Between Physical Activity and Plasma Biomarkers in the Cognitively Impaired Group **eTable 7.** Association Between Plasma Biomarkers and Cognition

Supplement 2.
Precision Medicine Platform for Mild Cognitive Impairment Based on Multi-omics, Imaging, Evidence-Based R&BD (PREMIER) Consortium Investigators

Supplement 3.
Data Sharing Statement
