# Supplementary material for: Physical Activity, Alzheimer Plasma Biomarkers, and Cognition
Source: JAMA Netw Open. 2025 Mar 5;8(3):e250096. doi: 10.1001/jamanetworkopen.2025.0096 (PMC11883494; doi:10.1001/jamanetworkopen.2025.0096)
Supplement: Supplement 1. — eMethods. eTable 1. Demographic Comparison Between Included and Excluded Participants eTable 2. Comparison Between Younger and Older Age Groups eTable 3. Association Between Physical Activity and Plasma Biomarkers in the Old Age (≥65) Group eTable 4. Association Between Physical Activity and Biomarkers in the Younger Age (<65) Group eTable 5. Association Between Physical Activity and Plasma Biomarkers in the Cognitively Unimpaired Group eTable 6. Association Between Physical Activity and Plasma Biomarkers in the Cognitively Impaired Group eTable 7. Association Between Plasma Biomarkers and Cognition [file jamanetwopen-e250096-s001.pdf]

Supplemental Online Content

Kim SA, Shin D, Ham H, et al; the Precision Medicine Platform for Mild Cognitive Impairment Based on Multi-omics, Imaging, Evidence-Based R&BD (PREMIER) Consortium. Physical activity, Alzheimer plasma biomarkers, and cognition. *JAMA Netw Open*. 2025;8(3):e250096. doi:10.1001/jamanetworkopen.2025.0096

eMethods.

eTable 1. Demographic Comparison Between Included and Excluded Participants

eTable 2. Comparison Between Younger and Older Age Groups

eTable 3. Association Between Physical Activity and Plasma Biomarkers in the Old Age (≥65) Group

eTable 4. Association Between Physical Activity and Biomarkers in the Younger Age (<65) Group

eTable 5. Association Between Physical Activity and Plasma Biomarkers in the Cognitively Unimpaired Group

eTable 6. Association Between Physical Activity and Plasma Biomarkers in the Cognitively Impaired Group

eTable 7. Association Between Plasma Biomarkers and Cognition

This supplemental material has been provided by the authors to give readers additional information about their work.

© 2025 Kim SA et al. *JAMA Network Open*.

eMethods

Physical Activity Assessment and Quantification

PA was evaluated using the International Physical Activity Questionnaire (IPAQ).[1] This tool measured the duration (time spent per day) and frequency (days per week) of different PA types, such as vigorous and moderate activities, walking, and sedentary behavior over the past seven days. The collected data were subsequently converted into Metabolic Equivalent Task minutes per week (MET-min/week) following the IPAQ scoring guidelines. MET minutes per week were calculated by multiplying the minutes spent on each activity per day by the number of days per week, and then by the respective MET value: 3.3 for walking, 4 for moderate activity, and 8 for vigorous activity.[1] In accordance with these guidelines, we limited the duration for each activity type to a maximum of 3 hours per day. Total MET was divided into four quartiles: 1st quartile (33.00≤total.MET<594.00; n=286), 2nd quartile (594.00≤total.MET<1386.00; n=286), 3rd quartile (1386.00≤total.MET<2747.25; n=286), and 4th quartile (2747.25≤total.MET≤15930.00; n=286).

Distribution of Total MET

© 2025 Kim SA et al. *JAMA Network Open*.

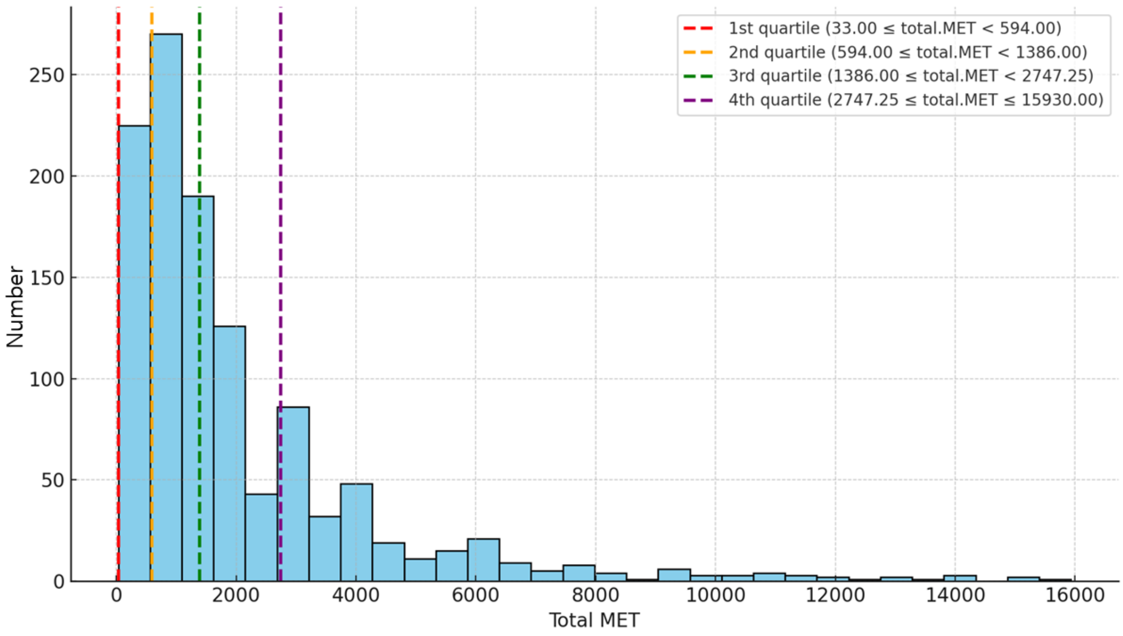

1st quartile (33.0≤total.MET<594.0) (n=286)

2nd quartile (594.0≤total.MET<1386.0) (n=286)

3rd quartile (1386.0≤total.MET<2747.3) (n=286)

4th quartile (2747.3≤total.MET≤15930.0) (n=286)

Plasma biomarkers collection and processing

Each participant provided 8 mL of blood, collected in a 0.5 M EDTA-containing tube and mixed for 5 minutes. Plasma was separated from the blood sample after centrifuging for 10 minutes at 1300×g and then aliquoted into five or ten vials of 0.3 mL each. Plasma samples were stored at -75 °C until analysis, following the guidelines set by the National Biobank of the Republic of Korea for human resource collection and registration. Frozen plasma samples were transported at -70 °C to the Department of Psychiatry and Neurochemistry, University of Gothenburg, for analysis. During analysis, samples were thawed on wet ice and centrifuged at 500×g for 5 minutes at 4 °C. Plasma levels of GFAP, and NfL were measured using the Neurology 4-Plex E kit (Quanterix, Billerica, MA, USA), while p-tau217 levels were measured using the ALZpath p-tau217 assay kit.

© 2025 Kim SA et al. *JAMA Network Open*.

Variable Assessments

Hypertension was defined as a reported history of diagnosis by a healthcare provider or the current use of antihypertensive medication. Diabetes Mellitus[2] was defined by either a reported history of diagnosis by a healthcare provider, the current use of antidiabetic medication, or an HbA1c level exceeding 6.5%. Body Mass Index (BMI) was calculated as weight in kilograms divided by the square of height in meters (kg/m^2). The Estimated Glomerular Filtration Rate (eGFR) was calculated using the Chronic Kidney Disease Epidemiology Collaboration equation[3], which incorporates age, sex, and serum creatinine levels.

Inclusion and Exclusion Criteria for PREMIER consortium

The study primarily focuses on mild cognitive impairment (MCI) and dementia due to Alzheimer's type (DAT), the most common forms of cognitive impairment in Korea, while also including a smaller number of frontotemporal dementia (FTD) cases. Normal controls (Cognitively Unimpaired, CU) include individuals visiting memory clinics nationwide for subjective cognitive complaints but determined to have normal ~~c~~cognition based on neuropsychological testing.

Inclusion Criteria:

General Criteria (for all participants):

1. Adults aged **40 years or older**.
2. Individuals (or their legal representatives) who voluntarily provided written informed consent after a full explanation of the study.

Specific Criteria:

© 2025 Kim SA et al. *JAMA Network Open*.

- **Cognitive Unimpaired (CU):**
    1. Neuropsychological test score at or above **mean – 1 SD** when adjusted for age, sex, and education level.
    2. Willing and able to complete all study procedures.
- **Amnestic Mild Cognitive Impairment (aMCI):**
    1. Cognitive complaints reported by the patient or caregiver.
    2. Neuropsychological tests showing impairment in cognitive abilities, including memory, relative to age and education.
    3. Preserved overall cognitive function.
    4. No significant impairment in daily activities.
    5. Clinical diagnosis confirms **no dementia**.
- **Dementia due to Alzheimer's type (DAT):**
    1. Diagnosis based on **NIA-AA diagnostic guidelines for probable AD dementia (core clinical criteria)**.
    2. MMSE score of **10 or higher**.
- **Frontotemporal Dementia (FTD):**
    1. Diagnosis includes bvFTD, nfvPPA, or svPPA based on specific diagnostic criteria:
        - bvFTD: **Bruce Miller et al. criteria.**[4]
        - svPPA and nfvPPA: **M.L. Gorno-Tempini et al. criteria.**[5]

Exclusion Criteria

Participants meeting any of the following criteria will be excluded:

1. Neurological abnormalities identified on medical examinations that may cause memory impairment.

© 2025 Kim SA et al. *JAMA Network Open*.

2. History of Axis I psychiatric disorders, including intellectual disability, schizophrenia, alcohol dependence, or bipolar disorder.
3. History of malignancy (cancer) within the past **3 years**. Exceptions: cervical carcinoma in situ or non-melanoma skin cancer.
4. History of brain surgery, carotid artery surgery, or other cerebrovascular operations.
5. Evidence of neurological symptoms suggestive of subcortical lesions, such as extrapyramidal signs indicative of cerebrovascular disease.
6. Presence of significant white matter changes on MRI, defined as:
    - Periventricular lesions: ≥10 mm parallel to the ventricles (extended cap) or irregular edges with deep white matter extension ≥10 mm (irregular halo).
    - Deep white matter lesions: confluent changes ≥25 mm or extensive deep white matter hyperintensities.
7. Shortness of breath at rest.
8. History of **cognitive, language, or problem-solving impairment lasting ≥2 hours** following a heart attack.
9. Psychiatric hospitalization within the past **5 years**.
10. History of substance abuse within the past **5 years**.
11. Treatment for alcohol dependence within the past **5 years**.
12. Loss of consciousness lasting **≥1 hour** not caused by general anesthesia.
13. Hospitalization due to head trauma.
14. Visual impairment preventing the ability to read standard text, even with corrective lenses.
15. Hearing impairment preventing comprehension of conversations, even with hearing aids.
16. Pregnant or breastfeeding women.
17. Any other condition deemed unsuitable for study participation by the investigator.

© 2025 Kim SA et al. *JAMA Network Open*.

eTable 1. Demographic comparison between included and excluded participants

Characteristic	Included participants (n= 1144)	Excluded participants (n=499)	*P*-value
**Age, y**	70.9 (8.7)	72.7 (8.8) (n=477)	<.001
**Female (%)**	744 (65.0)	314 (62.8) (n=314)	.42
**Education, y**	10.7 (4.7)	9.9 (4.8) (n= 465)	.001
**Diagnosis (%)**			
CU/MCI/DAT	256(22.4)/713(62.3)/175(15.3)	74(12.9)/347(60.2)/78(13.5)	<.001
**Vascular risk factors**			
Hypertension (%)	562 (49.1)	228 (45.6)	.21
Diabetes Mellitus (%)	270 (23.6)	121 (24.2)	.84
eGFR	88.4 (15.2)	85.9 (17.2) (n= 446)	.006
Body Mass Index	23.6 (3.3)	23.6 (3.2) (n= 418)	.80
**APOE4, carrier (%)**	406 (35.5)	167 (33.4)	.45
**Aβ uptake (rdcCL)**	45.6 (55.1)	40.3 (52.2) (n= 344)	.10
**Physical activity (MET-min/week)**	2059.3 (2297.6)	1842.8 (1827.8) (n= 206)	.13
**MMSE**	24.7 (4.5)	23.9 (4.9) (n= 456)	.004
**CDR-SB**	1.9 (2.1)	2.1 (2.3) (n= 449)	.06

Abbreviations: CU, cognitively unimpaired; MCI, mild cognitive impairment; DAT, dementia due to Alzheimer's type; eGFR, estimated glomerular filtration rate; rdcCL, regional direct conversion centiloid; MET, Metabolic Equivalent Task minutes per week; MMSE, Mini-Mental State Examination; CDR-SB, Clinical Dementia Rating-Sum of Boxes

[a]Values are presented as the mean (standard deviation) or number (%), as appropriate.

© 2025 Kim SA et al. *JAMA Network Open*.

eTable 2. Comparison between younger and older age group

Characteristic	Age < 65 (N=268)	Age ≥ 65 (N=876)	P value
**Age, y**	58.6 (4.7)	74.6 (5.7)	<.001
**Sex, female (%)**	182 (67.9)	562 (64.2)	.29
**Education, years**	12.5 (3.8)	10.2 (4.8)	<.001
**APOE ε4, carrier**	83 (31.0)	323 (36.9)	.09
**Diagnosis (%)**			
CU/MCI/DAT	75(28.0)/132(49.3)/61(22.8)	181(20.7)/581(66.3)/114(13.0)	<.001
**Physical activity, MET**	2342.7 (2446.4)	1972.7 (2244.4)	.03
**Aβ uptake (rdcCL)**	36.5 (54.0)	48.3 (55.1)	.002
**Plasma biomarkers (%)**			
Aβ42/40	0.1 (0.01)	0.1 (0.02)	.019
Ptau217	0.6 (0.6)	0.7 (0.6)	.001
GFAP	106.7 (62.7)	148.5 (81.9)	<.001
NfL	19.3 (10.5)	33.0 (26.6)	<.001
**Vascular risk factors**			
Hypertension (%)	91 (34.0)	471 (53.8)	<.001
Diabetes Mellitus (%)	31 (11.6)	239 (27.3)	<.001
eGFR	99.5 (11.9)	85.0 (14.4)	<.001
Body Mass Index	23.5 (3.0)	23.7 (3.4)	.35
**Cognition**			
MMSE	25.4 (4.8)	24.4 (4.3)	.005
CDR-SB	2.0 (2.8)	1.8 (1.9)	.31

Abbreviations: CU, cognitively unimpaired; MCI, mild cognitive impairment; DAT, dementia due to Alzheimer's type; MET, Metabolic Equivalent Task minutes per week; rdcCL, regional direct conversion centiloid; PTau217, phosphorylated tau 217; GFAP, glial fibrillary acidic protein; NfL, neurofilament light chain; eGFR, estimated glomerular filtration rate; MMSE, Mini-Mental State Examination; CDR-SB, Clinical Dementia Rating-Sum of Boxes

[a]Values are presented as the mean (standard deviation) or number (%), as appropriate.

© 2025 Kim SA et al. *JAMA Network Open*.

eTable 3. Association between physical activity and plasma biomarkers in the old age (≥ 65) group (N = 876)

Biomarkers	Q1	Q2		Q3		Q4		*P* for trend
		Estimate (SE)	*p* value	Estimate (SE)	*p* value	Estimate (SE)	*p* value	
**Aβ42/40**								
**Crude**	*reference*	0.002 (0.04)	.97	0.03 (0.04)	.43	0.05 (0.04)	.25	.18
**Model 1**	*reference*	-0.002 (0.04)	.96	0.03 (0.04)	.43	0.05 (0.04)	.27	.20
**Model 2**	*reference*	-0.01 (0.04)	.73	0.01 (0.04)	.75	0.02 (0.04)	.69	.57
**Model 3**	*reference*	-0.01 (0.04)	.77	0.02 (0.04)	.65	0.02 (0.04)	.64	.51
**Ptau 217**								
**Crude**	*reference*	-0.16 (0.10)	.12	-0.19 (0.10)	.072	-0.40 (0.11)	<.001	<.001
**Model 1**	*reference*	-0.13 (0.10)	.19	-0.16 (0.10)	.12	-0.32 (0.11)	.003	.004
**Model 2**	*reference*	-0.08 (0.06)	.22	-0.06 (0.06)	.34	-0.16 (0.07)	.02	.03
**Model 3**	*reference*	-0.06 (0.06)	.31	-0.03 (0.06)	.59	-0.16 (0.06)	.01	.03
**GFAP**								
**Crude**	*reference*	-0.06 (0.07)	.35	-0.03 (0.07)	.66	-0.31 (0.07)	<.001	<.001
**Model 1**	*reference*	-0.03 (0.07)	.60	0.02 (0.07)	.81	-0.18 (0.07)	.01	.04
**Model 2**	*reference*	-0.01 (0.06)	.84	0.06 (0.06)	.31	-0.11 (0.06)	.06	.22
**Model 3**	*reference*	0.004 (0.06)	.95	0.07 (0.06)	.21	-0.12 (0.06)	.05	.18
**NfL**								
**Crude**	*reference*	-0.14 (0.07)	.038	-0.16 (0.07)	.017	-0.27(0.07)	<.001	<.001
**Model 1**	*reference*	-0.10 (0.06)	.10	-0.13 (0.06)	.038	-0.17 (0.06)	.009	.008
**Model 2**	*reference*	-0.09 (0.06)	.12	-0.11 (0.06)	.059	-0.15 (0.06)	.02	.02
**Model 3**	*reference*	-0.07 (0.05)	.18	-0.08 (0.06)	.17	-0.13 (0.06)	.02	.03

Model 1: Adjusted for age and sex
Model 2: Further adjusted for Aβ uptakes
Model 3: Further adjusted for hypertension, diabetes, eGFR, and BMI
Physical activity was measured as Metabolic Equivalent Task minutes per week (MET-min/week) using the International Physical Activity questionnaire, MET was categorized as quartiles (lowest, Q1 – highest Q4)
Abbreviations: Q, quartile; SE, standard error; PTau217, phosphorylated tau 217; GFAP, glial fibrillary acidic protein; NfL, neurofilament light chain; eGFR, estimated glomerular filtration rate; BMI, body mass index;

© 2025 Kim SA et al. *JAMA Network Open*.

eTable 4. Association between physical activity and biomarkers in the younger age (< 65) group (N = 268)

Biomarkers	Q1	Q2 Estimate (SE)	Q2 *p* value	Q3 Estimate (SE)	Q3 *p* value	Q4 Estimate (SE)	Q4 *p* value	*P* for trend
**Aβ42/40**								
**Crude**	*reference*	-0.09 (0.07)	.17	0.03 (0.06)	.66	0.01 (0.06)	.93	.49
**Model 1**	*reference*	-0.08 (0.07)	.26	0.06 (0.07)	.35	0.04 (0.06)	.55	.22
**Model 2**	*reference*	-0.07 (0.06)	.23	0.04 (0.06)	.51	0.03 (0.05)	.59	.26
**Model 3**	*reference*	-0.07 (0.06)	.25	0.04 (0.06)	.51	0.03 (0.06)	.59	.26
**Ptau 217**								
**Crude**	*reference*	0.12 (0.23)	.62	-0.19 (0.22)	.39	-0.02 (0.21)	.92	.64
**Model 1**	*reference*	0.06 (0.23)	.80	-0.26 (0.23)	.26	-0.09 (0.21)	.68	.46
**Model 2**	*reference*	0.03 (0.11)	.80	-0.11 (0.11)	.31	-0.04 (0.10)	.69	.49
**Model 3**	*reference*	0.03 (0.11)	.79	-0.11 (0.11)	.33	-0.06 (0.10)	.55	.36
**GFAP**								
**Crude**	*reference*	0.20 (0.15)	.20	-0.01 (0.15)	.93	-0.01 (0.14)	.93	.56
**Model 1**	*reference*	0.17 (0.16)	.27	-0.01 (0.15)	.97	-0.01 (0.14)	.92	.60
**Model 2**	*reference*	0.15 (0.12)	.19	0.07 (0.12)	.56	0.01 (0.11)	.92	.78
**Model 3**	*reference*	0.13 (0.11)	.25	0.08 (0.11)	.48	-0.03 (0.10)	.78	.55
**NfL**								
**Crude**	*reference*	0.04 (0.12)	.72	-0.12 (0.12)	.29	0.02 (0.11)	.89	.83
**Model 1**	*reference*	-0.02 (0.12)	.84	-0.19 (0.12)	.10	-0.06 (0.11)	.57	.41
**Model 2**	*reference*	-0.03 (0.11)	.77	-0.16 (0.11)	.14	-0.05 (0.10)	.62	.50
**Model 3**	*reference*	-0.03 (0.10)	.80	-0.15 (0.10)	.14	-0.08 (0.10)	.42	.31

Model 1: Adjusted for age and sex
Model 2: Further adjusted for Aβ uptakes
Model 3: Further adjusted for hypertension, diabetes, eGFR, and BMI
Physical activity was measured as Metabolic Equivalent Task minutes per week (MET-min/week) using the International Physical Activity questionnaire, MET was categorized as quartiles (lowest, Q1 – highest Q4)
Abbreviations: Q, quartile; SE, standard error; PTau217, phosphorylated tau 217; GFAP, glial fibrillary acidic protein; NfL, neurofilament light chain; eGFR, estimated glomerular filtration rate; BMI, body mass index;

© 2025 Kim SA et al. *JAMA Network Open*.

eTable 5. Association between physical activity and plasma biomarkers in the CU group (N=256)

Biomarkers	Q1	Q2 Estimate (SE)	Q2 *p* value	Q3 Estimate (SE)	Q3 *p* value	Q4 Estimate (SE)	Q4 *p* value	*P* for trend
**Aβ42/40**								
**Crude**	*reference*	-0.07 (0.07)	.32	0.06 (0.07)	.35	0.004 (0.07)	.95	.41
**Model 1**	*reference*	-0.06 (0.07)	.34	0.07 (0.07)	.29	0.01 (0.07)	.94	.40
**Model 2**	*reference*	-0.01 (0.06)	.90	0.08 (0.06)	.21	0.03 (0.07)	.68	.37
**Model 3**	*reference*	-0.01 (0.06)	.84	0.08 (0.06)	.20	0.03 (0.07)	.62	.32
**GFAP**								
**Crude**	*reference*	0.11 (0.13)	.40	0.10 (0.13)	.44	-0.09 (0.13)	.49	.43
**Model 1**	*reference*	0.11 (0.11)	.32	0.12 (0.11)	.29	0.01 (0.12)	.92	.97
**Model 2**	*reference*	0.01 (0.10)	.92	0.10 (0.10)	.33	-0.03 (0.11)	.79	.97
**Model 3**	*reference*	0.02 (0.10)	.85	0.11 (0.10)	.26	-0.03 (0.10)	.75	.99
**Ptau217**								
**Crude**	*reference*	0.12 (0.16)	.45	-0.05 (0.16)	.75	-0.11 (0.16)	.48	.25
**Model 1**	*reference*	0.10 (0.14)	.48	-0.08 (0.14)	.55	-0.11 (0.15)	.45	.21
**Model 2**	*reference*	-0.13 (0.10)	.19	-0.12 (0.10)	.22	-0.21 (0.10)	.05	.08
**Model 3**	*reference*	-0.13 (0.10)	.17	-0.10 (0.09)	.30	-0.20 (0.10)	.04	.08
**NfL**								
**Crude**	*reference*	0.04 (0.13)	.74	-0.13 (0.13)	.29	-0.07 (0.13)	.58	.29
**Model 1**	*reference*	0.02 (0.10)	.83	-0.17 (0.10)	.09	-0.05 (0.10)	.61	.24
**Model 2**	*reference*	-0.02 (0.10)	.83	-0.17 (0.10)	.07	-0.07 (0.10)	.49	.23
**Model 3**	*reference*	-0.04 (0.09)	.69	-0.14 (0.09)	.10	-0.05 (0.09)	.61	.38

Model 1: age, sex
Model 2: age, sex, Aβ uptakes
Model 3: age, sex, Aβ uptakes, hypertension, diabetes, eGFR, and BMI
Physical activity was measured as Metabolic Equivalent Task minutes per week (MET-min/week) using the International Physical Activity questionnaire, MET was categorized as quartiles (lowest, Q1 – highest Q4)
Abbreviations: CU, cognitively unimpaired; Q, quartile; SE, standard error; PTau217, phosphorylated tau 217; GFAP, glial fibrillary acidic protein; NfL, neurofilament light chain; eGFR, estimated glomerular filtration rate; BMI, body mass index;

© 2025 Kim SA et al. *JAMA Network Open*.

eTable 6. Association between physical activity and plasma biomarkers in the CI group (N=888)

Biomarkers	Q1	Q2 Estimate (SE)	Q2 *p* value	Q3 Estimate (SE)	Q3 *p* value	Q4 Estimate (SE)	Q4 *p* value	*P* for trend
**Aβ42/40**								
**Crude**	*reference*	-0.02 (0.04)	.65	0.004 (0.04)	.92	0.05 (0.04)	.23	.20
**Model 1**	*reference*	-0.02 (0.04)	.68	0.01 (0.04)	.81	0.05 (0.04)	.21	.18
**Model 2**	*reference*	-0.03 (0.04)	.47	-0.003 (0.04)	.93	0.02 (0.04)	.51	.44
**Model 3**	*reference*	-0.02 (0.04)	.50	0.001 (0.04)	.99	0.03 (0.04)	.48	.40
**GFAP**								
**Crude**	*reference*	0.01 (0.07)	.87	-0.02 (0.07)	.76	-0.31 (0.07)	<.001	<.001
**Model 1**	*reference*	0.01 (0.07)	.87	0.02 (0.07)	.79	-0.17 (0.07)	.02	.03
**Model 2**	*reference*	0.04 (0.06)	.56	0.05 (0.06)	.40	-0.11 (0.06)	.08	.13
**Model 3**	*reference*	0.03 (0.06)	.55	0.07 (0.06)	.26	-0.12 (0.06)	.05	.10
**Ptau217**								
**Crude**	*reference*	-0.08 (0.11)	.48	-0.14 (0.11)	.19	-0.35 (0.11)	.001	.001
**Model 1**	*reference*	-0.08 (0.11)	.45	-0.13 (0.11)	.24	-0.28 (0.11)	.01	.01
**Model 2**	*reference*	-0.03 (0.07)	.66	-0.06 (0.07)	.38	-0.13 (0.07)	.05	.05
**Model 3**	*reference*	-0.03 (0.06)	.67	-0.03 (0.06)	.61	-0.14 (0.06)	.04	.05
**NFL**								
**Crude**	*reference*	-0.10 (0.07)	.19	-0.13 (0.07)	.08	-0.29 (0.07)	<.001	<.001
**Model 1**	*reference*	-0.10 (0.06)	.10	-0.11 (0.06)	.07	-0.17 (0.06)	.008	.009
**Model 2**	*reference*	-0.10 (0.06)	.12	-0.10 (0.06)	.10	-0.15 (0.06)	.02	.02
**Model 3**	*reference*	-0.09 (0.06)	.11	-0.07 (0.06)	.25	-0.15 (0.06)	.009	.02

Model 1: age, sex
Model 2: age, sex, Aβ uptakes
Model 3: age, sex, Aβ uptakes, hypertension, diabetes, eGFR, and BMI
Physical activity was measured as Metabolic Equivalent Task minutes per week (MET-min/week) using the International Physical Activity questionnaire, MET was categorized as quartiles (lowest, Q1 – highest Q4)
Abbreviations: CI, cognitively impaired; Q, quartile; SE, standard error; PTau217, phosphorylated tau 217; GFAP, glial fibrillary acidic protein; NfL, neurofilament light chain; eGFR, estimated glomerular filtration rate; BMI, body mass index;

© 2025 Kim SA et al. *JAMA Network Open*.

eTable 7. Association between plasma biomarkers and cognition

	Aβ42/40		PTau217		GFAP		NfL	
**Cognition**	Estimate (SE)	*p* value	Estimate (SE)	*p* value	Estimate (SE)	*p* value	Estimate (SE)	*p* value
**MMSE**	1.53 (0.30)	<.001	-1.60 (0.10)	<.001	-2.10 (0.15)	<.001	-1.80 (0.18)	<.001
**CDR-SB**	-0.94 (0.15)	<.001	0.84 (0.05)	<.001	1.05 (0.08)	<.001	0.96 (0.09)	<.001

Adjusted for age, sex, education years
Abbreviations: PTau217, phosphorylated tau 217; GFAP, glial fibrillary acidic protein; NfL, neurofilament light chain; SE, standard error; MMSE, Mini-Mental State Examination; CDR-SB, Clinical Dementia Rating-Sum of Boxes;

© 2025 Kim SA et al. *JAMA Network Open*.

References

1. Craig CL, Marshall AL, Sjöström M, et al. International physical activity questionnaire: 12-country reliability and validity. 2003;35(8):1381-1395.

2. Albert MS, DeKosky ST, Dickson D, et al. The diagnosis of mild cognitive impairment due to Alzheimer's disease: recommendations from the National Institute on Aging-Alzheimer's Association workgroups on diagnostic guidelines for Alzheimer's disease. *Alzheimer's & dementia : the journal of the Alzheimer's Association*. May 2011;7(3):270-9. doi:10.1016/j.jalz.2011.03.008

3. Inker LA, Eneanya ND, Coresh J, et al. New Creatinine- and Cystatin C-Based Equations to Estimate GFR without Race. *The New England journal of medicine*. Nov 4 2021;385(19):1737-1749. doi:10.1056/NEJMoa2102953

4. Rascovsky K, Hodges JR, Knopman D, et al. Sensitivity of revised diagnostic criteria for the behavioural variant of frontotemporal dementia. *Brain : a journal of neurology*. Sep 2011;134(Pt 9):2456-77. doi:10.1093/brain/awr179

5. Gorno-Tempini ML, Hillis AE, Weintraub S, et al. Classification of primary progressive aphasia and its variants. *Neurology*. Mar 15 2011;76(11):1006-14. doi:10.1212/WNL.0b013e31821103e6

© 2025 Kim SA et al. *JAMA Network Open*.
