# Supplementary material for: Physical Activity, Alzheimer Plasma Biomarkers, and Cognition
Source: JAMA Netw Open. 2025 Mar 5;8(3):e250096. doi: 10.1001/jamanetworkopen.2025.0096 (PMC11883494; doi:10.1001/jamanetworkopen.2025.0096)
Supplement: Supplement 2. — Precision Medicine Platform for Mild Cognitive Impairment Based on Multi-omics, Imaging, Evidence-Based R&BD (PREMIER) Consortium Investigators [file jamanetwopen-e250096-s002.pdf]

***Group Name(s): Precision Medicine Platform for Mild Cognitive Impairment Based on Multi-omics, Imaging, Evidence-Based R&BD (PREMIER) Consortium Investigators**

*First Name and	*Last Name	*Suffix	Academic	Institution	Location (city,	Role or Contribution,	Group (if more than 1
Sang Won	Seo		MD.PhD	Samsung Medical Center	Seoul, South Korea	Principal Investigator	PREMIER consortium
Duk L.	Na		MD.PhD	Samsung Medical Center	Seoul, South Korea	Principal Investigator	PREMIER consortium
Hyemin	Jang		MD.PhD	Seoul National University Hospital	Seoul, South Korea	Executive Committee	PREMIER consortium
Youngsoo	Kim		PhD	CHA University	Seongnam, South Korea	Executive Committee	PREMIER consortium
Sun-Ho	Han		PhD	Seoul National University	Seoul, South Korea	Executive Committee	PREMIER consortium
JoonKyung	Seong		PhD	Korea University	Seoul, South Korea	Executive Committee	PREMIER consortium
Jun-Kyu	Choi		PhD	Small Machines Company, Ltd.	Seoul, South Korea	Executive Committee	PREMIER consortium
Eek-Sung	Lee		MD.PhD	Soonchunhyang University Buche	Bucheon, South Korea	Executive Committee	PREMIER consortium
Juhee	Chin		PhD	Samsung Medical Center	Seoul, South Korea	Executive Committee	PREMIER consortium
Chi-Hun	Kim		MD.PhD	Hallym University Sacred Heart H	Anyang, South Korea	Executive Committee	PREMIER consortium
Hee Jin	Kim		MD.PhD	Samsung Medical Center	Seoul, South Korea	Executive Committee	PREMIER consortium
Haesook	Bok		MS	Samsung Medical Center	Seoul, South Korea	Executive Committee	PREMIER consortium
Sang Won	Seo		MD.PhD	Samsung Medical Center	Seoul, South Korea	Clinical Cohort Inves	PREMIER consortium
Duk L.	Na		MD.PhD	Samsung Medical Center	Seoul, South Korea	Clinical Cohort Inves	PREMIER consortium
Hyemin	Jang		MD.PhD	Seoul National University Hospital	Seoul, South Korea	Clinical Cohort Inves	PREMIER consortium
Hee Jin	Kim		MD.PhD	Samsung Medical Center	Seoul, South Korea	Clinical Cohort Inves	PREMIER consortium
Sung Hoon	Kang		MD.PhD	Department of Neurology, Korea U	Seoul, South Korea	Clinical Cohort Inves	PREMIER consortium
Yeshin	Kim		MD.PhD	Kangwon National University Hosp	Chuncheon, South Korea	Clinical Cohort Inves	PREMIER consortium
Chi-Hun	Kim		MD.PhD	Hallym University Sacred Heart H	Anyang, South Korea	Clinical Cohort Inves	PREMIER consortium
Si Eun	Kim		MD	Inje University Haeundae Paik Ho	Busan, South Korea	Clinical Cohort Inves	PREMIER consortium
Hang-Rai	Kim		MD.PhD	Dongguk University Ilsan Hospital	Goyang, South Korea	Clinical Cohort Inves	PREMIER consortium
Na-Yeon	Jung		MD.PhD	Pusan National University Yangsa	Yangsan, South Korea	Clinical Cohort Inves	PREMIER consortium
Seung Joo	Kim		MD	Gyeongsang National University C	Changwon, South Korea	Clinical Cohort Inves	PREMIER consortium
Seunghee	Na		MD	Incheon St. Mary's Hospital, the C	Incheon, South Korea	Clinical Cohort Inves	PREMIER consortium
Geon Ha	Kim		MD.PhD	Ewha Womans University, College	Seoul, South Korea	Clinical Cohort Inves	PREMIER consortium
Ko Woon	Kim		MD.PhD	Department of Neurology, Jeonbu	Jeonju, South Korea	Clinical Cohort Inves	PREMIER consortium
Jin San	Lee		MD.PhD	Kyung Hee University	Seoul, South Korea	Clinical Cohort Inves	PREMIER consortium
Hanna	Cho		MD.PhD	Gangnam Severance Hospital, Yo	Seoul, South Korea	Clinical Cohort Inves	PREMIER consortium
Yeo Jin	Kim		MD.PhD	Kangdong Sacred Heart Hospital	Seoul, South Korea	Clinical Cohort Inves	PREMIER consortium
Soo Hyun	Cho		MD.PhD	Department of Neurology, Chonna	Gwangju, South Korea	Clinical Cohort Inves	PREMIER consortium
Byeong C.	Kim		MD.PhD	Department of Neurology, Chonna	Gwangju, South Korea	Clinical Cohort Inves	PREMIER consortium
Dong Young	Lee		MD.PhD	Seoul National University Hospital	Seoul, South Korea	Clinical Cohort Inves	PREMIER consortium
So Young	Moon		MD.PhD	Department of Neurology, Ajou Ur	Suwon, South Korea	Clinical Cohort Inves	PREMIER consortium

Min Soo	Byun		MD.PhD	Seoul National University Hospital	Seoul, South Korea	Clinical Cohort Inves	PREMIER consortium
Giijung	Jung		RN.PhD	Seoul National University Hospital	Seoul, South Korea	Clinical Cohort Inves	PREMIER consortium
Dahyun	Yi		PhD	Seoul National University Hospital	Seoul, South Korea	Clinical Cohort Inves	PREMIER consortium
Han Na	Lee		RN	Seoul National University Hospital	Seoul, South Korea	Clinical Cohort Inves	PREMIER consortium
Jae-Won	Jang		MD.PhD	Kangwon National University Hos	Chuncheon, South Kore	Clinical Cohort Inves	PREMIER consortium
Eek-Sung	Lee		MD.PhD	Soonchunhyang University Buche	Bucheon, South Korea	Clinical Cohort Inves	PREMIER consortium
Jee Hyang	Jeong		MD.PhD	Ewha Womans University Seoul H	Seoul, South Korea	Clinical Cohort Inves	PREMIER consortium
Young Hee	Jung		MD.PhD	Myongji Hospital, College of Medi	Goyang, South Korea	Clinical Cohort Inves	PREMIER consortium
Jong Hun	Kim		MD.PhD	Ilsan Hospital, National Health Ins	Goyang, South Korea	Clinical Cohort Inves	PREMIER consortium
Young	Noh		MD.PhD	Gil Medical Center, Gachon Unive	Incheon, South Korea	Clinical Cohort Inves	PREMIER consortium
Hyunjung	Yang		CCRC	Pusan National University Yangsa	Yangsan, South Korea	Clinical Cohort Inves	PREMIER consortium
Youngji	Ha		RN.CCRC	Pusan National University Yangsa	Yangsan, South Korea	Clinical Cohort Inves	PREMIER consortium
Hae-Eun	Shin		-	Bucheon St. Mary's Hospital, the (	Bucheon, South Korea	Clinical Cohort Inves	PREMIER consortium
Kyunghun	Kang		MD.PhD	Department of Neurology, School	Daegu, South Korea	Clinical Cohort Inves	PREMIER consortium
SungHui	Eom		bachelor's	Pusan National University Yangsa	Yangsan, South Korea	Clinical Cohort Inves	PREMIER consortium
Juhee	Chin		Ph.D	Samsung Medical Center	Seoul, South Korea	eCRF Core	PREMIER consortium
Haesook	Bok		MS	Samsung Medical Center	Seoul, South Korea	eCRF Core	PREMIER consortium
Youngsoo	Kim		PhD.	CHA University	Seongnam, South Korea	Blood Biomarker Dev	PREMIER consortium
Sun-Ho	Han		PhD	Seoul National University	Seoul, South Korea	Blood Biomarker Dev	PREMIER consortium
Ki Young	Shin		PhD	Seoul National University	Seoul, South Korea	Blood Biomarker Dev	PREMIER consortium
Yeongshin	Kim		PhD.	CHA University	Seongnam, South Korea	Blood Biomarker Dev	PREMIER consortium
Jisung	Jang		PhD.	Quantamatrix Inc.	Seoul, South Korea	Blood Biomarker Dev	PREMIER consortium
Changsik	Yoon		MS	Quantamatrix	Seoul, South Korea	Blood Biomarker Dev	PREMIER consortium
Do kyung	Lee		MS	Quantamatrix	Seoul, South Korea	Blood Biomarker Dev	PREMIER consortium
JoonKyung	Seong		Ph.D	Korea University	Seoul, South Korea	Imaging Core	PREMIER consortium
Hongki	Ham		master's d	Samsung Medical Center	Seoul, South Korea	Imaging Core	PREMIER consortium
Yu Hyun	Park		PhD.	Samsung Medical Center	Seoul, South Korea	Imaging Core	PREMIER consortium
Soo-Jong	Kim		MS	Samsung Medical Center	Seoul, South Korea	Imaging Core	PREMIER consortium
Byunghyun	Byun		MD.PhD	Korea Institute of Radiological anc	Seoul, South Korea	Imaging Core	PREMIER consortium
Yejoo	Choi		M.S.	Samsung Medical Center	Seoul, South Korea	CSF Core	PREMIER consortium
Na Kyung	Lee		Ph.D.	Samsung Medical Center	Seoul, South Korea	CSF Core	PREMIER consortium
Hong-Hee	Won		Ph.D.	Sungkyunkwan University, Samsu	Seoul, South Korea	Genomic Core	PREMIER consortium
Minyoung	Cho		MS.	Sungkyunkwan University	Suwon, South Korea	Genomic Core	PREMIER consortium
Sang-Hyuk	Jung		PhD.	University of Pennsylvania	Philadelphia, PA, USA	Genomic Core	PREMIER consortium
Dong Hyun	Lee		MD.	Oneomics	Seoul, South Korea	Genomic Core	PREMIER consortium
Beomsu	Kim		B.S.	Sungkyunkwan University	Suwon, South Korea	Genomic Core	PREMIER consortium

Jun-Kyu	Choi		PhD.	Small Machines Company, Ltd.	Seoul, South Korea	Software	PREMIER consortium
Jinkyu	Seo		None	Small Machines	Seoul, South Korea	Software	PREMIER consortium
Bo Kyoung	Cheon		PhD.	Sungkyunkwan University	Suwon, South Korea	Data Management	PREMIER consortium
Youngju	Kim		MA	Samsung Medical Center	Seoul, South Korea	Data Management	PREMIER consortium
