# Supplementary material for: Physical Activity, Alzheimer Plasma Biomarkers, and Cognition
Source: JAMA Netw Open. 2025 Mar 5;8(3):e250096. doi: 10.1001/jamanetworkopen.2025.0096 (PMC11883494; doi:10.1001/jamanetworkopen.2025.0096)
Supplement: Supplement 3. — Data Sharing Statement [file jamanetwopen-e250096-s003.pdf]

Data Sharing Statement

Kim. Physical Activity, Alzheimer Plasma Biomarkers, and Cognition. *JAMA Netw Open*. Published March 05, 2025. doi:10.1001/jamanetworkopen.2025.0096

Data
Data available: No

Additional Information
Explanation for why data not available: Reason for unavailability: The datasets generated and analyzed in this study are not publicly accessible due to ethical concerns and privacy restrictions. However, data may be requested from the corresponding author, pending approval from the Institutional Review Board.
